# Large deflection analysis of circular piezoelectric micro-actuator with flexoelectric effect

**DOI:** 10.1038/s41598-023-45990-8

**Published:** 2023-11-08

**Authors:** Xue Ji

**Affiliations:** Department of Industrial Engineering, School of Management Engineering, Shandong Jianzhu University, Jinan, 250101 People’s Republic of China

**Keywords:** Materials science, Mathematics and computing, Physics

## Abstract

At micro/nano scale, the stiffening effect and flexoelectric effect of strain gradient play important roles in the electromechanical coupling response of piezoelectric micro-components. In this paper, the large deflection bending problem of circular piezoelectric micro-actuator is studied based on the extended linear dielectric theory. In addition to the piezoelectric effect, the flexoelectric effect, the stiffening effect of strain gradient and the high-order electric field effect of polarization gradient are introduced. According to the variational principle, a size-dependent model of circular piezoelectric micro-actuator is established to investigate its electromechanical coupling response. The contributions of piezoelectric effect and flexoelectric effect on large deflection behaviors of piezoelectric micro-actuator are revealed. It is hoped that the research results will be helpful to further understand the electromechanical coupling properties of piezoelectric micro-components and improve the control precision of piezoelectric micro-actuator.

## Introduction

Piezoelectric micro-actuator is the core component of micro-electromechanical system (MEMS) which can convert electrical energy into mechanical energy by the electromechanical coupling effect. The micro-displacement system composed of piezoelectric actuator has been widely used in the fields of ultra-precision machining, microelectronics system and even organs-on-chips for its small size, high efficiency, high displacement resolution and no noise. Up to now, the static and dynamic characteristics of piezoelectric actuators have been studied in great depth. Vilarinho et al.^1^ reported an engineering case study of piezoelectric actuators in gas microvalves and established the relations between applied voltages, bender displacements, gas pressure drops through the microvalve and associated flow rates. The static displacement of a three-layer axisymmetric circular piezoelectric unimorph actuator subjected to voltage and uniform pressure loads has been investigated by Dereshgi et al.^2^. Przybylski and Kuliński^3^ revealed the deformation and nonlinear free vibrations control of a sandwich piezoelectric beam system under piezoelectric actuation. A nonlinear model has been proposed by Ascione et al.^4^ for buckling, postbuckling and nonlinear static response analyses of geometrically imperfect composite beams with piezoelectric actuators. Reddy et al.^5^ investigated the nonlinear dynamics and active control of smart beams using the shear mode and extensional mode of piezoelectric actuators. However, these studies are based on traditional theories. In fact, the mechanical properties of the components at the micro scale are obviously different from those at the macro scale.

Mcfarland and Colton^6^ observed in the bending experiments of 15 and 30 micron thick polypropylene cantilever beams that the bending stiffness was more than 4 times of the traditional theoretical predicted value. Lam et al.^7^ observed in the bending experiment of an epoxy resin cantilever beam that the dimensionless bending stiffness of a 20 micron thick microbeam increased by about 2.3 times compared with that of a 115 micron microbeam. The traditional theory cannot explain the dependence of micro-component deformation behavior on the characteristic size, but the relevant research shows that the size effect phenomena can be described well by strain gradient theory^8–11^. Based on the modified strain gradient theory, a microstructure-dependent Timoshenko piezoelectric beam model has been presented by Li and Feng^12^. Jafari et al.^13^ analyzed the free vibration of rectangular microplates with bonded piezoelectric layers based on the modified couple stress theory. The nonlinear response of functionally graded piezoelectric beam actuator has been investigated by Komijani et al.^14^ based on the modified couple stress theory and the Timoshenko beam theory with the von Kármán nonlinearity. Shahrokhi et al.^15^ utilized the modified couple stress theory to consider the size effect and studied the vibrational behaviors of sandwich piezoelectric micro-plate. Hai et al.^16^ investigated the vibrational behavior of a sandwich honeycomb rectangular microplate integrated with piezoelectric actuators and rested on the Pasternak elastic foundation based on the modified strain gradient theory.

Furthermore, flexoelectric effect plays an important role in the electromechanical coupling phenomena at micro- and nano-scale. Flexoelectric effect is an inherent electromechanical coupling effect of all dielectrics in which strain gradient can induce polarization (direct flexoelectric effect) and, vice versa, polarization gradient can generate mechanical stress (inverse flexoelectric effect)^17,18^. The flexoelectricity-induced polarization can be significantly increased as structures are scaled down due to the scaling effect of strain gradient^19^. An enhancement in the piezoelectric effect of up to 70% has been revealed by Qi et al. in the local probing of the buckled PZT ribbons^20^. A substantial piezoelectric response has been measured well above the Curie temperature in the reduced PZT ceramic wafers due to flexoelectric effect^21^. The experimental work carried through at Penn State to explore the flexoelectric coefficients in ferroelectric, incipient ferroelectric and relaxor ferroelectric perovskites has been summarized by Cross^22^. Zhang et al.^23^ investigated the 2312 flexoelectric coefficient component of polyvinylidene fluoride. In order to improve the dielectric and flexoelectric properties of the BST films, different concentrations of K^+^ and Mg^2+^ have been doped by Dong et al.^24^. An enhanced flexoelectricity in Al_2_O_3_-doped Ba(Ti_0.85_Sn_0.15_)O_3_ ceramics has been reported by Shu et al.^25^ in which the transverse flexoelectric coefficient of the 0.5 wt% Al_2_O_3_-doped ceramic is almost 2 times larger than that of pure Ba(Ti_0.85_Sn_0.15_)O_3_ ceramic.

In order to capture the flexoelectric effect, the extended linear theory for dielectrics has been presented, in which strain gradient and polarization gradient are also considered in addition to the traditional strain and polarization^26–28^. Based on the higher-order theory, Chen et al.^29^ analyzed the dynamic response of piezoelectric and flexoelectric Euler–Bernoulli beam. Wang and Li^30^ studied the electromechanical coupling responses of nanoplates with the piezoelectric and flexoelectric effects and found that the flexoelectric effect is thickness-dependent. Zeng et al.^31^ investigated the nonlinear vibration of piezoelectric sandwich nanoplates with functionally graded porous core by considering piezoelectric effect, flexoelectric effect and von Karman type large deformation. Chen and Yan^32^ proposed a nonlinear electromechanical model for energy harvester based on axially preloaded piezoelectric beam incorporating flexoelectric effect. Deng et al.^33^ examined flexoelectric energy harvesting under harmonic mechanical excitation and found the output power density and conversion efficiency increase significantly when the beam thickness reduces from micro to nanoscale. The nonlinear vibration of a functionally graded flexoelectric energy harvesting nanobeams has been analyzed by Chu et al.^34^. In addition, PN heterojunctions associated bending coupling in flexoelectric semiconductor composites have also been studied by Li et al.^35^.

In this work, the nonlinear bending behavior of piezoelectric micro-actuator with flexoelectric effect is investigated. A size-dependent model of piezoelectric micro-actuator of axisymmetric circular plate with flexoelectric effect is presented in section “Model of a piezoelectric micro-actuator of axisymmetric circular plate with flexoelectric effect”. The corresponding governing equations and boundary conditions are derived based on variational principle. The differential quadrature method (DQM) is applied to solve the nonlinear bending questions of micro-actuator under simply supported boundary condition and clamped boundary condition and the large deflection bending behaviors of piezoelectric micro-actuator is revealed in section “Large deflection bending behaviors of circular piezoelectric micro-actuator with flexoelectric effect”, respectively. Finally, conclusions are summarized in section “Conclusions”.

## Model of a piezoelectric micro-actuator of axisymmetric circular plate with flexoelectric effect

**Figure 1:**
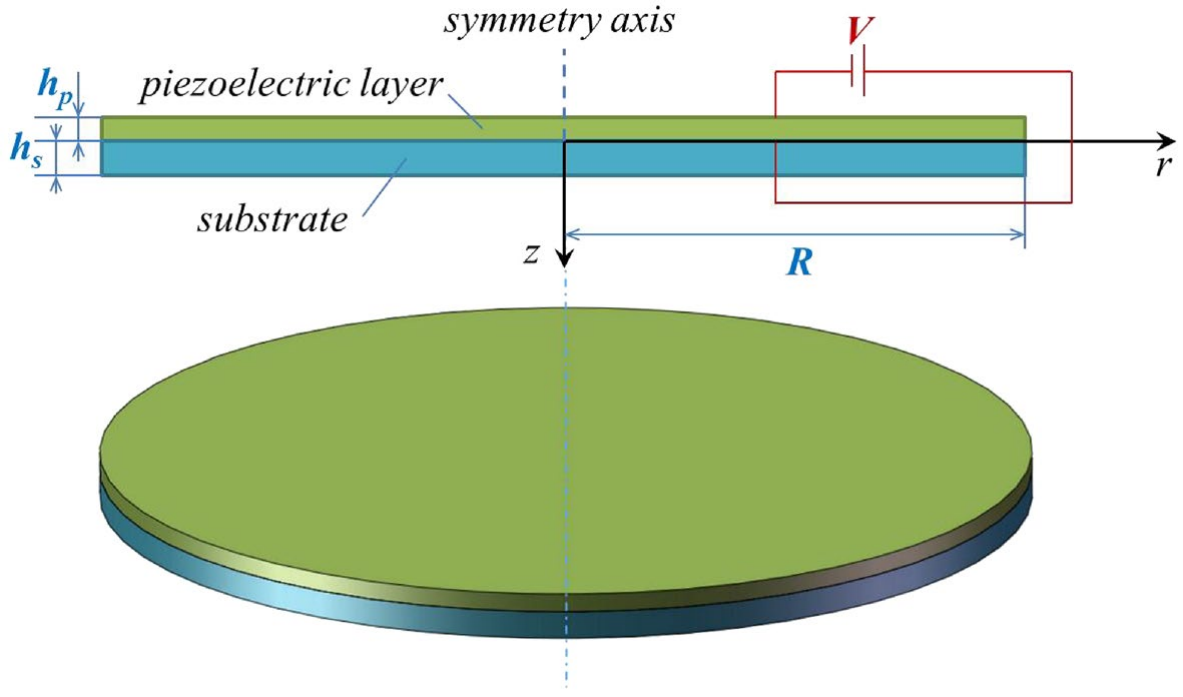
Sketch of piezoelectric micro-actuator of axisymmetric circular plate.

Consider a piezoelectric micro-actuator of axisymmetric circular plate, as shown in Fig. 1, which consists of a substrate and a piezoelectric layer bonded on the surface of the substrate. The thicknesses of substrate and piezoelectric layer are *h*_*s*_ and *h*_*p*_, and it should be noted here that the subscripts *s* and *p* represent the substrate and piezoelectric layer, respectively. The radius of the circular plate is *R*. A cylindrical coordinate is adopted to model the piezoelectric micro-actuator, in which the *r*–*θ* plane coincides with the interface of the substrate and piezoelectric layer. The driving voltage applied to the upper and lower surfaces of the piezoelectric layer is denoted as *V*.

According to the Kirchhoff hypothesis, the deformation displacements of the present circular plate can be described as

1 $$ u_{r} (r,z) = u(r) - z\frac{\partial w}{{\partial r}},\quad u_{\theta } (r,z) = 0,\quad u_{z} (r,z) = w(r) $$

where *u*_*r*_, *u*_*θ*_ and *u*_*z*_ are displacement components along the *r*-, *θ*- and *z*- directions respectively and *u* represents the radial displacement at the interface of the substrate and piezoelectric layer. According to the Von Kármán’s strain theory, the large strain components are

2 $$ \varepsilon_{rr} = \frac{\partial u}{{\partial r}} + \frac{1}{2}\left( {\frac{\partial w}{{\partial r}}} \right)^{2} - z\frac{{\partial^{2} w}}{{\partial r^{2} }},\quad \varepsilon_{\theta \theta } = \frac{u}{r} - \frac{z}{r}\frac{\partial w}{{\partial r}} $$

Then the corresponding strain gradient are given by

3 $$ \varepsilon_{rr,z} = - \frac{{\partial^{2} w}}{{\partial r^{2} }},\quad \varepsilon_{\theta \theta ,z} = - \frac{\partial w}{{r\partial r}} $$

where a comma denotes differentiation with respect to the coordinates. It should be noted here that the strain gradients along the radial direction are neglected compared to that along the thickness direction for the current thin circular plate due to the much smaller thickness than its radius. Thus, for the flexoelectric effect, only that induced by the strain gradient along the thickness direction is considered in this work. In addition, the polarization is assumed to be along the thickness direction only, expressed as

4 $$ P_{z} = P_{z} (r,z) $$

And, similarly, only the polarization gradient along the thickness direction is considered in this work.

In order to incorporate the flexoelectric effect, an extended linear theory of dielectrics is applied in this paper, which also includes the coupling of strain gradient to strain gradient, the coupling of polarization gradient to polarization gradient, the coupling of strain gradient to polarization and the coupling of polarization gradient to strain in comparison with the piezoelectric theory. The internal energy *U* is expressed as^28^

5 $$ U = \int_{\Omega } {\left[ {\frac{1}{2}c_{ijkl} \varepsilon_{ij} \varepsilon_{kl} + \frac{1}{2}a_{ij} P_{i} P_{j} + d_{ijk} P_{i} \varepsilon_{jk} + \frac{1}{2}g_{ijklmn} \varepsilon_{jk,i} \varepsilon_{mn,l} + \frac{1}{2}b_{ijkl} P_{i,j} P_{k,l} + f_{ijkl} (P_{i} \varepsilon_{kl,j} - P_{i,j} \varepsilon_{kl} )} \right]} {\text{d}}\Omega $$

in which *ε*_*ij*_ and *P*_*i*_ are the strain tensor and polarization vector, respectively. *c*_*ijkl*_, *a*_*ij*_ and *d*_*ijk*_ are the elastic constant, reciprocal dielectric susceptibility and piezoelectric constant tensors, respectively. The material property tensors *g*_*ijklmn*_ and *b*_*ijkl*_ stand for the higher-order elastic effect and electric field effect, respectively, and *f*_*ijkl*_ represents the flexocoupling coefficient tensor. The present theory considers the piezoelectric effect, the flexoelectric effect, the stiffening effect of strain gradient and the high-order electric field effect of polarization gradient, and can reduce to other simplified theory by deleting certain effects. For example, when the terms associated with strain gradient and polarization gradient are ignored, the present theory will reduce to the traditional piezoelectric theory. If the stiffening effect of strain gradient is preserved, the piezoelectric strain gradient theory can be obtained. The present theory can also reduce to the flexoelectric theory of centrosymmetric materials by deleting the piezoelectric effect. When the terms associated with polarization are neglected, the strain gradient theory can be obtained and the internal energy *U* in Eq. (5) will reduce to^10^

6 $$ U_{s} = \int_{\Omega } {\left( {\frac{1}{2}c_{ijkl} \varepsilon_{ij} \varepsilon_{kl} + \frac{1}{2}g_{ijklmn} \varepsilon_{jk,i} \varepsilon_{mn,l} } \right)} {\text{d}}\Omega $$

Moreover, the contracted notation for the subscripts of the material property tensors is adopted for simplicity, i.e., *c*_11_ = *c*_1111_, *c*_12_ = *c*_1122_, *g*_11_ = *g*_311311_, *g*_12_ = *g*_311322_, *d*_311_ = *d*_31_, *f*_31_ = *f*_3311_, *b*_33_ = *b*_3333_.

For a transversely isotropic piezoelectric circular plate, the internal energy of piezoelectric layer, *U*_*p*_, can be obtained by substituting Eqs. (2)–(4) into Eq. (5), given by

7 $$ \begin{aligned} U_{p} = & 2\pi \int_{{ - h_{p} }}^{0} {\int_{0}^{R} {\left[ {\frac{1}{2}c_{11}^{p} \left( {\varepsilon_{rr} \varepsilon_{rr} + \varepsilon_{\theta \theta } \varepsilon_{\theta \theta } } \right) + c_{12}^{p} \varepsilon_{rr} \varepsilon_{\theta \theta } + \frac{1}{2}g_{11}^{p} \left( {\varepsilon_{rr,z} \varepsilon_{rr,z} + \varepsilon_{\theta \theta ,z} \varepsilon_{\theta \theta ,z} } \right) + g_{12}^{p} \varepsilon_{rr,z} \varepsilon_{\theta \theta ,z} } \right.} } \\ & + \left. {\frac{1}{2}a_{33} P_{z} P_{z} + \frac{1}{2}b_{33} \left( {\frac{{\partial P_{z} }}{\partial z}} \right)^{2} + d_{31} P_{z} \left( {\varepsilon_{rr} + \varepsilon_{\theta \theta } } \right) + f_{31} \left[ {P_{z} (\eta_{zrr} + \eta_{z\theta \theta } ) - \frac{{\partial P_{z} }}{\partial z}(\varepsilon_{rr} + \varepsilon_{\theta \theta } )} \right]} \right]rdrdz \\ \end{aligned} $$

and the internal energy of elastic substrate, *U*_*s*_, from Eq. (6) is expressed as

8 $$ U_{s} = 2\pi \int_{0}^{{h_{s} }} {\int_{0}^{R} {\left[ {\frac{1}{2}c_{11}^{s} (\varepsilon_{rr} \varepsilon_{rr} + \varepsilon_{\theta \theta } \varepsilon_{\theta \theta } ) + c_{12}^{s} \varepsilon_{rr} \varepsilon_{\theta \theta } + \frac{1}{2}g_{11}^{s} (\varepsilon_{rr,z} \varepsilon_{rr,z} + \varepsilon_{\theta \theta ,z} \varepsilon_{\theta \theta ,z} ) + g_{12}^{s} \varepsilon_{rr,z} \varepsilon_{\theta \theta ,z} } \right]} } rdrdz $$

in which the superscripts *s* and *p* in elastic constants and higher-order elastic constants represent the substrate and piezoelectric layer, respectively. Furthermore, the total electric enthalpy *H* is given by

9 $$ \begin{aligned} H & = U_{p} + U_{s} + 2\pi \int_{{ - h_{p} }}^{0} {\int_{0}^{R} {\left[ { - \frac{1}{2}\varepsilon_{0} \varphi_{,z} \varphi_{,z} + \varphi_{,z} P_{z} } \right]rdrdz} } \\ & = 2\pi \int_{0}^{R} {S(r)} rdr + 2\pi \int_{{ - h_{p} }}^{0} {\int_{0}^{R} {\left[ {\frac{1}{2}a_{33} P_{z} P_{z} + \frac{1}{2}b_{33} \left( {\frac{{\partial P_{z} }}{\partial z}} \right)^{2} + d_{31} P_{z} \left( {\frac{u}{r} + \frac{\partial u}{{\partial r}} + \frac{1}{2}\left( {\frac{\partial w}{{\partial r}}} \right)^{2} - z\left( {\frac{{\partial^{2} w}}{{\partial r^{2} }} + \frac{\partial w}{{r\partial r}}} \right)} \right)} \right.} } \\ & \quad - \left. {f_{31} \left[ {P_{z} \left( {\frac{{\partial^{2} w}}{{\partial r^{2} }} + \frac{\partial w}{{r\partial r}}} \right) + \frac{{\partial P_{z} }}{\partial z}\left( {\frac{u}{r} + \frac{\partial u}{{\partial r}} + \frac{1}{2}\left( {\frac{\partial w}{{\partial r}}} \right)^{2} - z\left( {\frac{{\partial^{2} w}}{{\partial r^{2} }} + \frac{\partial w}{{r\partial r}}} \right)} \right)} \right] - \frac{1}{2}\varepsilon_{0} \varphi_{,z} \varphi_{,z} + \varphi_{,z} P_{z} } \right]rdrdz \\ \end{aligned} $$

where *φ* is the potential of the Maxwell self-field, *ε*_0_ is the permittivity of a vacuum and *S*(*r*) is shown as Eq. (A.1) in Appendix A.

Consider the work done by a transverse load *q*(*r*), $W = 2\pi \int_{0}^{R} {qwr{\text{d}}r}$, and further according to the variational principle, $\delta ( - H + W) = 0$, the electrical governing equations can be derived as

10 $$ a_{33} P_{z} - b_{33} \frac{{\partial^{2} P_{z} }}{{\partial z^{2} }} + \varphi_{,z} + d_{31} \left( {\frac{u}{r} + \frac{\partial u}{{\partial r}} + \frac{1}{2}\left( {\frac{\partial w}{{\partial r}}} \right)^{2} } \right) - d_{31} z\left( {\frac{{\partial^{2} w}}{{\partial r^{2} }} + \frac{\partial w}{{r\partial r}}} \right) - 2f_{31} \left( {\frac{{\partial^{2} w}}{{\partial r^{2} }} + \frac{\partial w}{{r\partial r}}} \right) = 0 $$

11 $$ - \varepsilon_{0} \varphi_{,zz} + P_{z,z} = 0 $$

and the electrical boundary conditions are written as

12 $$ \left[ {b_{33} \frac{{\partial P_{z} }}{\partial z} - f_{31} \left( {\frac{u}{r} + \frac{\partial u}{{\partial r}} + \frac{1}{2}\left( {\frac{\partial w}{{\partial r}}} \right)^{2} - z\left( {\frac{{\partial^{2} w}}{{\partial r^{2} }} + \frac{\partial w}{{r\partial r}}} \right)} \right)} \right]\delta P_{z} |_{{ - h_{p} }}^{0} = 0 $$

13 $$ \left( { - \varepsilon_{0} \varphi_{,z} + P_{z} } \right)\delta \varphi |_{{ - h_{p} }}^{0} = 0 $$

For the piezoelectric micro-actuator subjected to a driving voltage *V* between its upper and lower surfaces, the electrical boundary conditions are

14 $$ b_{33} \frac{{\partial P_{z} }}{\partial z} - \left. {f_{31} \left( {\frac{u}{r} + \frac{\partial u}{{\partial r}} + \frac{1}{2}\left( {\frac{\partial w}{{\partial r}}} \right)^{2} - z\left( {\frac{{\partial^{2} w}}{{\partial r^{2} }} + \frac{\partial w}{{r\partial r}}} \right)} \right)} \right|_{{z = - h_{p} \,or\,0}} = 0\quad \varphi |_{z = 0} = V\quad \varphi |_{{z = - h_{p} }} = 0 $$

Combining the electrical governing equations Eqs. (10) and (11) and boundary conditions Eq. (14), the polarization and electric potential can be solved, respectively, as

15 $$ \begin{aligned} P_{z} = & \left( {\frac{{f_{31} }}{{\lambda b_{33} }}\frac{{e^{{\lambda (h_{p} /2 + z)}} - e^{{ - \lambda (h_{p} /2 + z)}} }}{{e^{{\lambda h_{p} /2}} + e^{{ - \lambda h_{p} /2}} }} - \frac{{d_{31} }}{{a_{33} }}} \right)\left( {\frac{u}{r} + \frac{\partial u}{{\partial r}} + \frac{1}{2}\left( {\frac{\partial w}{{\partial r}}} \right)^{2} } \right) - \frac{1}{{b_{33} \lambda^{2} }}\left[ {\frac{{d_{31} }}{\lambda }\frac{{e^{{\lambda (h_{p} /2 + z)}} - e^{{ - \lambda (h_{p} /2 + z)}} }}{{e^{{\lambda h_{p} /2}} + e^{{ - \lambda h_{p} /2}} }}} \right. \\ & + \left. {\lambda f_{31} h_{p} \frac{{e^{\lambda z} + e^{ - \lambda z} }}{{e^{{\lambda h_{p} }} - e^{{ - \lambda h_{p} }} }} - d_{31} z - \frac{{2f_{31} (1 + 2\varepsilon_{0} a_{33} ) - d_{31} h_{p} }}{{2\varepsilon_{0} a_{33} }}} \right]\left( {\frac{\partial w}{{r\partial r}} + \frac{{\partial^{2} w}}{{\partial r^{2} }}} \right) - \frac{V}{{a_{33} h_{p} }} \\ \end{aligned} $$

16 $$ \begin{aligned} \varphi = & \frac{{f_{31} }}{{\varepsilon_{0} b_{33} \lambda^{2} }}\left( {\frac{{e^{{\lambda (h_{p} /2 + z)}} + e^{{ - \lambda (h_{p} /2 + z)}} }}{{e^{{\lambda h_{p} /2}} + e^{{ - \lambda h_{p} /2}} }} - 1} \right)\left( {\frac{u}{r} + \frac{\partial u}{{\partial r}} + \frac{1}{2}\left( {\frac{\partial w}{{\partial r}}} \right)^{2} } \right) - \frac{1}{{\varepsilon_{0} b_{33} \lambda^{2} }}\left[ {\frac{{d_{31} }}{{\lambda^{2} }}\frac{{e^{{\lambda (h_{p} /2 + z)}} + e^{{ - \lambda (h_{p} /2 + z)}} }}{{e^{{\lambda h_{p} /2}} + e^{{ - \lambda h_{p} /2}} }}} \right. \\ & + \left. {f_{31} h_{p} \frac{{e^{\lambda z} - e^{ - \lambda z} }}{{e^{{\lambda h_{p} }} - e^{{ - \lambda h_{p} }} }} - \frac{{d_{31} }}{2}z^{2} - \left( {f_{31} + \frac{{d_{31} h_{p} }}{2}} \right)z - \frac{{d_{31} }}{{\lambda^{2} }}} \right]\left( {\frac{\partial w}{{r\partial r}} + \frac{{\partial^{2} w}}{{\partial r^{2} }}} \right) + \left( {\frac{z}{{h_{p} }} + 1} \right)V \\ \end{aligned} $$

in which the parameter *λ* is defined as

17 $$ \lambda = \sqrt {\frac{{\varepsilon_{0} a_{33} + 1}}{{\varepsilon_{0} b_{33} }}} $$

The mechanical governing equation can be further derived by considering the solution of polarization and electric potential as

18 $$ \begin{aligned} & \left( {c_{11}^{s} h_{s} + c_{11}^{p} h_{p} - t_{3} } \right)\left( {\frac{u}{{r^{2} }} - \frac{\partial u}{{r\partial r}} - \frac{{\partial^{2} u}}{{\partial r^{2} }} - \frac{\partial w}{{\partial r}}\frac{{\partial^{2} w}}{{\partial r^{2} }}} \right) + \left[ {c_{12}^{p} h_{p} + c_{12}^{s} h_{s} - \left( {c_{11}^{s} h_{s} + c_{11}^{p} h_{p} } \right)} \right]\frac{1}{2r}\left( {\frac{\partial w}{{\partial r}}} \right)^{2} \\ & \quad + \left[ { - \frac{1}{2}\left( {c_{11}^{p} h_{p}^{2} - c_{11}^{s} h_{s}^{2} } \right) + t_{2} } \right]\left( {\frac{{\partial^{3} w}}{{\partial r^{3} }} + \frac{{\partial^{2} w}}{{r\partial r^{2} }} - \frac{\partial w}{{r^{2} \partial r}}} \right) = 0 \\ \end{aligned} $$

19 $$ \begin{aligned} & - \left[ {\frac{1}{3}\left( {c_{11}^{s} h_{s}^{3} + c_{11}^{p} h_{p}^{3} } \right) + g_{11}^{p} h_{p} + g_{11}^{s} h_{s} + t_{1} } \right]\nabla^{4} w + \left( {c_{11}^{s} h_{s} + c_{11}^{p} h_{p} - t_{3} } \right)\left[ {\frac{1}{2r}\left( {\frac{\partial w}{{\partial r}}} \right)^{3} + \frac{3}{2}\left( {\frac{\partial w}{{\partial r}}} \right)^{2} \frac{{\partial^{2} w}}{{\partial r^{2} }} + \frac{\partial u}{{\partial r}}\frac{\partial w}{{r\partial r}} + \frac{{\partial^{2} u}}{{\partial r^{2} }}\frac{\partial w}{{\partial r}}} \right. \\ & \quad + \left. {\frac{\partial u}{{\partial r}}\frac{{\partial^{2} w}}{{\partial r^{2} }}} \right] + \left( {c_{12}^{p} h_{p} + c_{12}^{s} h_{s} - t_{3} } \right)\left( {\frac{\partial u}{{\partial r}}\frac{\partial w}{{r\partial r}} + \frac{u}{r}\frac{{\partial^{2} w}}{{\partial r^{2} }}} \right) - \left[ {\frac{1}{2}\left( {c_{11}^{p} h_{p}^{2} - c_{11}^{s} h_{s}^{2} } \right) - t_{2} } \right]\left( {\frac{u}{{r^{3} }} - \frac{\partial u}{{r^{2} \partial r}} + 2\frac{{\partial^{2} u}}{{r\partial r^{2} }} + \frac{{\partial^{3} u}}{{\partial r^{3} }}} \right) \\ & \quad - \left[ {\frac{1}{2}\left( {c_{11}^{p} h_{p}^{2} - c_{11}^{s} h_{s}^{2} } \right) - \frac{3}{2}\left( {c_{12}^{p} h_{p}^{2} - c_{12}^{s} h_{s}^{2} } \right) + 2t_{2} } \right]\frac{\partial w}{{r\partial r}}\frac{{\partial^{2} w}}{{\partial r^{2} }} - \frac{{d_{31} V}}{{a_{33} }}\left( {\frac{\partial w}{{r\partial r}} + \frac{{\partial^{2} w}}{{\partial r^{2} }}} \right) + q = 0 \\ \end{aligned} $$

and the mechanical boundary conditions are

20 $$ \begin{aligned} & r\left[ {\left( {c_{12}^{p} h_{p} + c_{12}^{s} h_{s} - t_{3} } \right)\frac{u}{r} + \left( {c_{11}^{s} h_{s} + c_{11}^{p} h_{p} - t_{3} } \right)\frac{\partial u}{{\partial r}} + \frac{1}{2}\left( {c_{11}^{s} h_{s} + c_{11}^{p} h_{p} - t_{3} } \right)\left( {\frac{\partial w}{{\partial r}}} \right)^{2} } \right. \\ & \quad + \left. {\frac{1}{2}(c_{11}^{p} h_{p}^{2} - c_{11}^{s} h_{s}^{2} - 2t_{2} )\frac{{\partial^{2} w}}{{\partial r^{2} }} + \frac{1}{2}\left( {c_{12}^{p} h_{p}^{2} - c_{12}^{s} h_{s}^{2} - 2t_{2} } \right)\frac{\partial w}{{r\partial r}} - \frac{{d_{31} V}}{{a_{33} }}} \right]\delta u|_{0}^{R} = 0 \\ \end{aligned} $$

21 $$ \begin{aligned} & r\left[ {\frac{1}{2}\left( {c_{11}^{p} h_{p}^{2} - c_{11}^{s} h_{s}^{2} - 2t_{2} } \right)\left( {\frac{u}{{r^{2} }} - \frac{\partial u}{{r\partial r}} - \frac{{\partial^{2} u}}{{\partial r^{2} }}} \right) + \left[ { - \frac{1}{4}\left( {c_{11}^{p} h_{p}^{2} - c_{11}^{s} h_{s}^{2} } \right) + \frac{3}{4}\left( {c_{12}^{p} h_{p}^{2} - c_{12}^{s} h_{s}^{2} } \right) - t_{2} } \right]\frac{1}{r}\left( {\frac{\partial w}{{\partial r}}} \right)^{2} } \right. \\ & \quad + \frac{1}{2}\left( {c_{11}^{s} h_{s} + c_{11}^{p} h_{p} - t_{3} } \right)\left( {\frac{\partial w}{{\partial r}}} \right)^{3} + \left[ {\frac{1}{3}\left( {c_{11}^{s} h_{s}^{3} + c_{11}^{p} h_{p}^{3} } \right) + g_{11}^{p} h_{p} + g_{11}^{s} h_{s} + t_{1} } \right]\left( {\frac{\partial w}{{r^{2} \partial r}} - \frac{{\partial^{2} w}}{{r\partial r^{2} }} - \frac{{\partial^{3} w}}{{\partial r^{3} }}} \right) \\ & \quad + \left. {\left( {c_{11}^{s} h_{s} + c_{11}^{p} h_{p} - t_{3} } \right)\frac{\partial u}{{\partial r}}\frac{\partial w}{{\partial r}} + \left( {c_{12}^{p} h_{p} + c_{12}^{s} h_{s} - t_{3} } \right)\frac{u}{r}\frac{\partial w}{{\partial r}} - \frac{{d_{31} V}}{{a_{33} }}\frac{\partial w}{{\partial r}}} \right]\delta w|_{0}^{R} = 0 \\ \end{aligned} $$

22 $$ \begin{aligned} & r\left[ {\frac{1}{2}\left( {c_{12}^{p} h_{p}^{2} - c_{12}^{s} h_{s}^{2} - 2t_{2} } \right)\frac{u}{r} + \frac{1}{2}\left( {c_{11}^{p} h_{p}^{2} - c_{11}^{s} h_{s}^{2} - 2t_{2} } \right)\frac{\partial u}{{\partial r}} + \frac{1}{4}\left( {c_{11}^{p} h_{p}^{2} - c_{11}^{s} h_{s}^{2} - 2t_{2} } \right)\left( {\frac{\partial w}{{\partial r}}} \right)^{2} } \right. \\ & \quad + \left[ {\frac{1}{3}\left( {c_{11}^{s} h_{s}^{3} + c_{11}^{p} h_{p}^{3} } \right) + g_{11}^{p} h_{p} + g_{11}^{s} h_{s} + t_{1} } \right]\frac{{\partial^{2} w}}{{\partial r^{2} }} + \left[ {\frac{1}{3}(c_{12}^{p} h_{p}^{3} + c_{12}^{s} h_{s}^{3} ) + g_{12}^{p} h_{p} + g_{12}^{s} h_{s} + t_{1} } \right]\frac{\partial w}{{r\partial r}} \\ & \quad + \left. {\frac{V}{{a_{33} }}\left( {f_{31} - \frac{{d_{31} h_{p} }}{2}} \right)} \right]\delta w_{,r} |_{0}^{R} = 0 \\ \end{aligned} $$

where $\nabla^{4} = \frac{{\partial^{4} }}{{\partial r^{4} }} + \frac{2}{r}\frac{{\partial^{3} }}{{\partial r^{3} }} - \frac{1}{{r^{2} }}\frac{{\partial^{2} }}{{\partial r^{2} }} + \frac{1}{{r^{3} }}\frac{\partial }{\partial r}$ and the coefficients *t*_*i*_(*i* = 1, 2, 3) are defined, respectively, as

23 $$ \begin{aligned} t_{1} = & \frac{2}{{b_{33} \lambda^{3} }}\left( {d_{31} f_{31} h_{p} - \frac{{d_{31}^{2} }}{{\lambda^{2} }}} \right)\frac{{e^{{\lambda h_{p} /2}} - e^{{ - \lambda h_{p} /2}} }}{{e^{{\lambda h_{p} /2}} + e^{{ - \lambda h_{p} /2}} }} - \frac{{f_{31}^{2} h_{p}^{2} }}{{b_{33} \lambda }}\frac{{e^{{\lambda h_{p} }} + e^{{ - \lambda h_{p} }} }}{{e^{{\lambda h_{p} }} - e^{{ - \lambda h_{p} }} }} \\ & + \frac{{h_{p} }}{{b_{33} \lambda^{2} }}\left( {f_{31}^{2} - \frac{3}{2}d_{31} f_{31} h_{p} + \frac{{d_{31}^{2} }}{{\lambda^{2} }} - \frac{{d_{31}^{2} h_{p}^{2} }}{3}} \right) + \frac{{2f_{31} d_{31} h_{p}^{2} (2 + 3\varepsilon_{0} a_{33} ) - d_{31}^{2} h_{p}^{3} }}{{4a_{33} (1 + \varepsilon_{0} a_{33} )}} - \frac{{f_{31}^{2} h_{p} }}{{a_{33} }} \\ \end{aligned} $$

24 $$ t_{2} = \frac{{f_{31} }}{{b_{33} \lambda^{2} }}\left( {\lambda f_{31} h_{p} - \frac{{2d_{31} }}{\lambda }} \right)\frac{{e^{{\lambda h_{p} /2}} - e^{{ - \lambda h_{p} /2}} }}{{e^{{\lambda h_{p} /2}} + e^{{ - \lambda h_{p} /2}} }} + \frac{{f_{31} d_{31} h_{p} }}{{b_{33} \lambda^{2} }} - \frac{{d_{31} h_{p} (2f_{31} - d_{31} h_{p} )}}{{2a_{33} }} $$

25 $$ t_{3} = \frac{{d_{31}^{2} h_{p} }}{{a_{33} }} + 2\frac{{f_{31}^{2} }}{{\lambda b_{33} }}\frac{{e^{{\lambda h_{p} /2}} - e^{{ - \lambda h_{p} /2}} }}{{e^{{\lambda h_{p} /2}} + e^{{ - \lambda h_{p} /2}} }} $$

The present model is a general model which includes piezoelectric effect, flexoelectric effect, the mechanical effect of strain gradient and the electrical effect of polarization gradient. When the piezoelectric effect is neglected by letting *d*_31_ = 0, the present model will reduce to that of flexoelectric theory. When the flexoelectric effect is neglected by letting *f*_31_ = 0, the present model will reduce to that of piezoelectric strain gradient theory.

Furthermore, the present model can be normalized by introducing dimensionless parameters,

26 $$ \begin{aligned} & \xi = \frac{r}{R},\quad \alpha = \frac{{h_{p} }}{h},\quad \vartheta = \frac{h}{R},\quad \overline{w} = \frac{w}{h},\quad \overline{u} = \frac{u}{h},\quad \overline{q}_{0} = \frac{2qR}{{\vartheta^{3} \left( {c_{11}^{s} h_{s} + c_{11}^{p} h_{p} - t_{3} } \right)}} \\ & \overline{V}_{p} = \frac{{2d_{31} V}}{{a_{33} \left( {c_{11}^{s} h_{s} + c_{11}^{p} h_{p} - t_{3} } \right)}},\quad \overline{V}_{f} = \frac{{2f_{31} V}}{{a_{33} h\left( {c_{11}^{s} h_{s} + c_{11}^{p} h_{p} - t_{3} } \right)}} \\ \end{aligned} $$

with *h* denoting the total thickness of micro-actuator and $h = h_{s} + h_{p}$. The dimensionless governing equations are written as

27 $$ \frac{{\overline{u}}}{{\xi^{2} }} - \frac{{\partial \overline{u}}}{\xi \partial \xi } - \frac{{\partial^{2} \overline{u}}}{{\partial \xi^{2} }} - \vartheta \frac{{\partial \overline{w}}}{\partial \xi }\frac{{\partial^{2} \overline{w}}}{{\partial \xi^{2} }} + k_{1} \vartheta \frac{1}{\xi }\left( {\frac{{\partial \overline{w}}}{\partial \xi }} \right)^{2} + k_{2} \left( {\frac{{\partial^{3} \overline{w}}}{{\partial \xi^{3} }} + \frac{{\partial^{2} \overline{w}}}{{\xi \partial \xi^{2} }} - \frac{{\partial \overline{w}}}{{\xi^{2} \partial \xi }}} \right) = 0 $$

28 $$ \begin{aligned} & - k_{3} \left( {\frac{{\partial^{4} \overline{w}}}{{\partial \xi^{4} }} + 2\frac{{\partial^{3} \overline{w}}}{{\xi \partial \xi^{3} }} - \frac{{\partial^{2} \overline{w}}}{{\xi^{2} \partial \xi^{2} }} + \frac{{\partial \overline{w}}}{{\xi^{3} \partial \xi }}} \right) + \frac{2}{\vartheta }\left( {\frac{{\partial^{2} \overline{u}}}{{\partial \xi^{2} }}\frac{{\partial \overline{w}}}{\partial \xi } + \frac{{\partial \overline{u}}}{\partial \xi }\frac{{\partial^{2} \overline{w}}}{{\partial \xi^{2} }} + \frac{{\partial \overline{u}}}{\partial \xi }\frac{{\partial \overline{w}}}{\xi \partial \xi }} \right) + \frac{1}{\xi }\left( {\frac{{\partial \overline{w}}}{\partial \xi }} \right)^{3} + 3\left( {\frac{{\partial \overline{w}}}{\partial \xi }} \right)^{2} \frac{{\partial^{2} \overline{w}}}{{\partial \xi^{2} }} \\ & \quad + \frac{{2k_{4} }}{\vartheta }\left( {\frac{{\partial \overline{u}}}{\partial \xi }\frac{{\partial \overline{w}}}{\xi \partial \xi } + \frac{{\overline{u}}}{\xi }\frac{{\partial^{2} \overline{w}}}{{\partial \xi^{2} }}} \right) - \frac{{k_{5} }}{\vartheta }\left( {\frac{{\overline{u}}}{{\xi^{3} }} - \frac{{\partial \overline{u}}}{{\xi^{2} \partial \xi }} + 2\frac{{\partial^{2} \overline{u}}}{{\xi \partial \xi^{2} }} + \frac{{\partial^{3} \overline{u}}}{{\partial \xi^{3} }}} \right) - k_{6} \frac{{\partial \overline{w}}}{\xi \partial \xi }\frac{{\partial^{2} \overline{w}}}{{\partial \xi^{2} }} \\ & \quad - \frac{1}{{\vartheta^{2} }}\overline{V}_{p} \left( {\frac{{\partial \overline{w}}}{\xi \partial \xi } + \frac{{\partial^{2} \overline{w}}}{{\partial \xi^{2} }}} \right) - \frac{1}{{\vartheta^{2} }}\frac{{\partial^{2} \overline{w}}}{{\partial \varsigma^{2} }} + \overline{q}_{0} = 0 \\ \end{aligned} $$

and the dimensionless boundary conditions are given by

29 $$ \xi \left[ {2k_{4} \frac{{\overline{u}}}{\xi } + 2\frac{{\partial \overline{u}}}{\partial \xi } + \vartheta \left( {\frac{{\partial \overline{w}}}{\partial \xi }} \right)^{2} + \vartheta k_{5} \frac{{\partial^{2} \overline{w}}}{{\partial \xi^{2} }} + \vartheta k_{7} \frac{{\partial \overline{w}}}{\xi \partial \xi } - \overline{V}_{p} } \right]\delta \overline{u}|_{0}^{1} = 0 $$

30 $$ \begin{aligned} & \xi \left[ {2\frac{{k_{2} }}{\vartheta }\left( { - \frac{{\overline{u}}}{{\xi^{2} }} + \frac{{\partial \overline{u}}}{\xi \partial \xi } + \frac{{\partial^{2} \overline{u}}}{{\partial \xi^{2} }}} \right) - \frac{\vartheta }{2}\frac{{k_{6} }}{\xi }\left( {\frac{{\partial \overline{w}}}{\partial \xi }} \right)^{2} + \vartheta \left( {\frac{{\partial \overline{w}}}{\partial \xi }} \right)^{3} + k_{3} \vartheta \left( {\frac{{\partial \overline{w}}}{{\xi^{2} \partial \xi }} - \frac{{\partial^{2} \overline{w}}}{{\xi \partial \xi^{2} }} - \frac{{\partial^{3} \overline{w}}}{{\partial \xi^{3} }}} \right)} \right. \\ & \quad + \left. {2\frac{{\partial \overline{u}}}{\partial \xi }\frac{{\partial \overline{w}}}{\partial \xi } + 2k_{4} \frac{{\overline{u}}}{\xi }\frac{{\partial \overline{w}}}{\partial \xi } - \frac{{\overline{V}_{p} }}{\vartheta }\frac{{\partial \overline{w}}}{\partial \xi }} \right]\delta \overline{w}|_{0}^{1} = 0 \\ \end{aligned} $$

31 $$ \xi \left[ {k_{7} \vartheta \frac{{\overline{u}}}{\xi } - 2k_{2} \frac{{\partial \overline{u}}}{\partial \xi } - k_{2} \vartheta \left( {\frac{{\partial \overline{w}}}{\partial \xi }} \right)^{2} + k_{3} \vartheta^{2} \frac{{\partial^{2} \overline{w}}}{{\partial \xi^{2} }} + 2k_{8} \vartheta^{2} \frac{{\partial \overline{w}}}{\xi \partial \xi } + \overline{V}_{f} - \frac{\alpha }{2}\overline{V}_{p} } \right]\delta \overline{w}_{,\xi } |_{0}^{1} = 0 $$

in which the dimensionless coefficients *k*_*i*_(*i* = 1, 2, …, 8) are shown as Eq. (A.2)–(A.9) in Appendix A.

## Large deflection bending behaviors of circular piezoelectric micro-actuator with flexoelectric effect

### Simply supported boundary condition

For the piezoelectric micro-actuator of axisymmetric circular plate with simply supported boundary conditions, the governing equations are shown as Eqs. (27) and (28). The boundary conditions are

32 $$ \overline{u}|_{\xi = 1} = 0,\quad \overline{w}|_{\xi = 1} = 0\quad \xi \left[ {k_{7} \vartheta \frac{{\overline{u}}}{\xi } - 2k_{2} \frac{{\partial \overline{u}}}{\partial \xi } - k_{2} \vartheta \left( {\frac{{\partial \overline{w}}}{\partial \xi }} \right)^{2} + k_{3} \vartheta^{2} \frac{{\partial^{2} \overline{w}}}{{\partial \xi^{2} }} + 2k_{8} \vartheta^{2} \frac{{\partial \overline{w}}}{\xi \partial \xi } + \overline{V}_{f} - \frac{1}{2}\alpha \overline{V}_{p} } \right]_{\xi = 1} = 0 $$

And the regularity conditions are written as

33 $$ \overline{u}|_{\xi = 0} = 0,\quad \overline{w}_{,\xi } |_{\xi = 0} = 0 $$

The nonlinear governing equations Eqs. (27) and (28) and boundary conditions Eqs. (32) and (33) can be solved by the differential quadrature method (DQM)^9^. The *k*-order partial derivatives with respect to *ξ* of a function *f*(*ξ*) at any sample point can be approximated by a weighted linear sum of the function values at all discrete points, shown as

34 $$ \frac{{\partial^{k} f(\xi_{i} )}}{{\partial \xi^{k} }} = \sum\limits_{j = 1}^{N} {A_{ij}^{(k)} f(\xi_{j} )} $$

in which *N* is the total number of discrete points and $A_{ij}^{(k)}$ is the *k*-order weighting coefficients matrix. According to the differential quadrature method, the governing equations are discretized as

35 $$ \begin{aligned} & \frac{{\overline{u}_{i} }}{{\xi_{i}^{2} }} - \frac{1}{{\xi_{i} }}\sum\limits_{j = 1}^{N} {A_{ij}^{(1)} \overline{u}_{j} } - \sum\limits_{j = 1}^{N} {A_{ij}^{(2)} \overline{u}_{j} } - \vartheta \sum\limits_{j = 1}^{N} {A_{ij}^{(1)} \overline{w}_{j} } \sum\limits_{j = 1}^{N} {A_{ij}^{(2)} \overline{w}_{j} } + k_{1} \vartheta \frac{1}{{\xi_{i} }}\sum\limits_{j = 1}^{N} {A_{ij}^{(1)} \overline{w}_{j} } \sum\limits_{j = 1}^{N} {A_{ij}^{(1)} \overline{w}_{j} } \\ & \quad + k_{2} \left( {\sum\limits_{j = 1}^{N} {A_{ij}^{(3)} \overline{w}_{j} } + \frac{1}{{\xi_{i} }}\sum\limits_{j = 1}^{N} {A_{ij}^{(2)} \overline{w}_{j} } - \frac{1}{{\xi_{i}^{2} }}\sum\limits_{j = 1}^{N} {A_{ij}^{(1)} \overline{w}_{j} } } \right) = 0 \\ \end{aligned} $$

36 $$ \begin{aligned} & - k_{3} \left( {\sum\limits_{j = 1}^{N} {A_{ij}^{(4)} \overline{w}_{j} } + \frac{2}{{\xi_{i} }}\sum\limits_{j = 1}^{N} {A_{ij}^{(3)} \overline{w}_{j} } - \frac{1}{{\xi_{i}^{2} }}\sum\limits_{j = 1}^{N} {A_{ij}^{(2)} \overline{w}_{j} } + \frac{1}{{\xi_{i}^{3} }}\sum\limits_{j = 1}^{N} {A_{ij}^{(1)} \overline{w}_{j} } } \right) \\ & \quad + \frac{2}{\vartheta }\left( {\sum\limits_{j = 1}^{N} {A_{ij}^{(2)} \overline{u}_{j} } \sum\limits_{j = 1}^{N} {A_{ij}^{(1)} \overline{w}_{j} } + \sum\limits_{j = 1}^{N} {A_{ij}^{(1)} \overline{u}_{j} } \sum\limits_{j = 1}^{N} {A_{ij}^{(2)} \overline{w}_{j} } + \frac{1}{{\xi_{i} }}\sum\limits_{j = 1}^{N} {A_{ij}^{(1)} \overline{u}_{j} } \sum\limits_{j = 1}^{N} {A_{ij}^{(1)} \overline{w}_{j} } } \right) \\ & \quad + \frac{1}{{\xi_{i} }}\sum\limits_{j = 1}^{N} {A_{ij}^{(1)} \overline{w}_{j} } \sum\limits_{j = 1}^{N} {A_{ij}^{(1)} \overline{w}_{j} } \sum\limits_{j = 1}^{N} {A_{ij}^{(1)} \overline{w}_{j} } + 3\sum\limits_{j = 1}^{N} {A_{ij}^{(1)} \overline{w}_{j} } \sum\limits_{j = 1}^{N} {A_{ij}^{(1)} \overline{w}_{j} } \sum\limits_{j = 1}^{N} {A_{ij}^{(2)} \overline{w}_{j} } \\ & \quad + \frac{{2k_{4} }}{\vartheta }\left( {\frac{1}{{\xi_{i} }}\sum\limits_{j = 1}^{N} {A_{ij}^{(1)} \overline{u}_{j} } \sum\limits_{j = 1}^{N} {A_{ij}^{(1)} \overline{w}_{j} } + \frac{{\overline{u}_{i} }}{{\xi_{i} }}\sum\limits_{j = 1}^{N} {A_{ij}^{(2)} \overline{w}_{j} } } \right) - \frac{{k_{5} }}{\vartheta }\left( {\frac{{\overline{u}_{i} }}{{\xi_{i}^{3} }} - \frac{1}{{\xi_{i}^{2} }}\sum\limits_{j = 1}^{N} {A_{ij}^{(1)} \overline{u}_{j} } + \frac{2}{{\xi_{i} }}\sum\limits_{j = 1}^{N} {A_{ij}^{(2)} \overline{u}_{j} } + \sum\limits_{j = 1}^{N} {A_{ij}^{(3)} \overline{u}_{j} } } \right) \\ & \quad - \frac{{k_{6} }}{{\xi_{i} }}\sum\limits_{j = 1}^{N} {A_{ij}^{(1)} \overline{w}_{j} } \sum\limits_{j = 1}^{N} {A_{ij}^{(2)} \overline{w}_{j} } - \frac{1}{{\vartheta^{2} }}\overline{V}_{p} \left( {\frac{1}{{\xi_{i} }}\sum\limits_{j = 1}^{N} {A_{ij}^{(1)} \overline{w}_{j} } + \sum\limits_{j = 1}^{N} {A_{ij}^{(2)} \overline{w}_{j} } } \right) = - \overline{q}_{0} \\ \end{aligned} $$

And the boundary conditions are

37 $$ \begin{aligned} & \overline{u}_{1} = 0,\quad \overline{u}_{N} = 0,\quad \sum\limits_{j = 1}^{N} {A_{1j}^{(1)} \overline{w}_{j} } = 0,\quad \overline{w}_{N} = 0 \\ & \quad k_{7} \vartheta \overline{u}_{N} - 2k_{2} \sum\limits_{j = 1}^{N} {A_{Nj}^{(1)} \overline{u}_{j} } - k_{2} \vartheta \sum\limits_{j = 1}^{N} {A_{Nj}^{(1)} \overline{w}_{j} \sum\limits_{j = 1}^{N} {A_{Nj}^{(1)} \overline{w}_{j} } } + k_{3} \vartheta^{2} \sum\limits_{j = 1}^{N} {A_{Nj}^{(2)} \overline{w}_{j} } + 2k_{8} \vartheta^{2} \sum\limits_{j = 1}^{N} {A_{Nj}^{(1)} \overline{w}_{j} } + \overline{V}_{f} - \frac{1}{2}\alpha \overline{V}_{p} = 0 \\ \end{aligned} $$

The discrete governing equations and boundary conditions Eqs. (35)–(37) can be written in matrix form

38 $$ ({\mathbf{K}}_{{\mathbf{L}}} + {\mathbf{K}}_{{{\mathbf{NL}}}} ){\mathbf{d}} = {\mathbf{F}} $$

where ${\mathbf{K}}_{{{\mathbf{NL}}}}$ and ${\mathbf{K}}_{{\mathbf{L}}}$ are equivalent nonlinear and linear stiffness matrices, ${\mathbf{F}}$ is the external force vector, ${\mathbf{d}} = \left\{ {\left\{ {\overline{u}_{i} } \right\},\left\{ {\overline{w}_{i} } \right\}} \right\}^{T} \,(i = 1,2, \ldots ,N)$ is the displacement vector. Finally, Eq. (38) can be soved by the iteration method.

Consider a polyvinylidene difluoride (PVDF) piezoelectric actuator in which PVDF is chosen as the piezoelectric layer and Polydimethylsiloxane (PDMS) is chosen as the substrate. The thickness of piezoelectric layer is set as 1.5 times the thickness of the substrate (*hp* = 1.5 hs) and the radius of the actuator is 25 times of the actuator thickness (*R* = 25 h). For PVDF, $c_{11}^{p} = 3.7\,{\text{GPa}}$, $c_{12}^{p} = 0.38 \cdot c_{11}^{p}$, *a*_33_ = 1.38 × 1010 Nm^2^/C^2^,* ε*_0_ = 8.854 × 10^−12^ F/m, *d*_31_ = − 1.0212 × 109 N/C, *f*_31_ = − 179 N m/C and, in addition, $\sqrt {{{g_{11}^{p} } \mathord{\left/ {\vphantom {{g_{11}^{p} } {c_{12}^{p} }}} \right. \kern-0pt} {c_{12}^{p} }}}$ is assumed to be 1 μm which is known as the material length scale parameter. For PDMS, $c_{11}^{s} = 2\,{\text{MPa}}$, $c_{12}^{s} = 0.38 \cdot c_{11}^{s}$ and its material length scale parameter is also assumed to be 1 μm. Therefore, the nonlinear bending of the present PVDF piezoelectric actuator subjected to a drive voltage *V* = 80 V are solved by applying DQM in which the normalized Gauss–Chebyshev–Lobatto points $\xi (i) = \frac{1}{2}[1 - \cos (\frac{i - 1}{{N - 1}}\pi )]$ ($i = 1,2, \ldots ,N$) are used to generate the DQM point system with setting *N* = 30.

**Figure 2:**
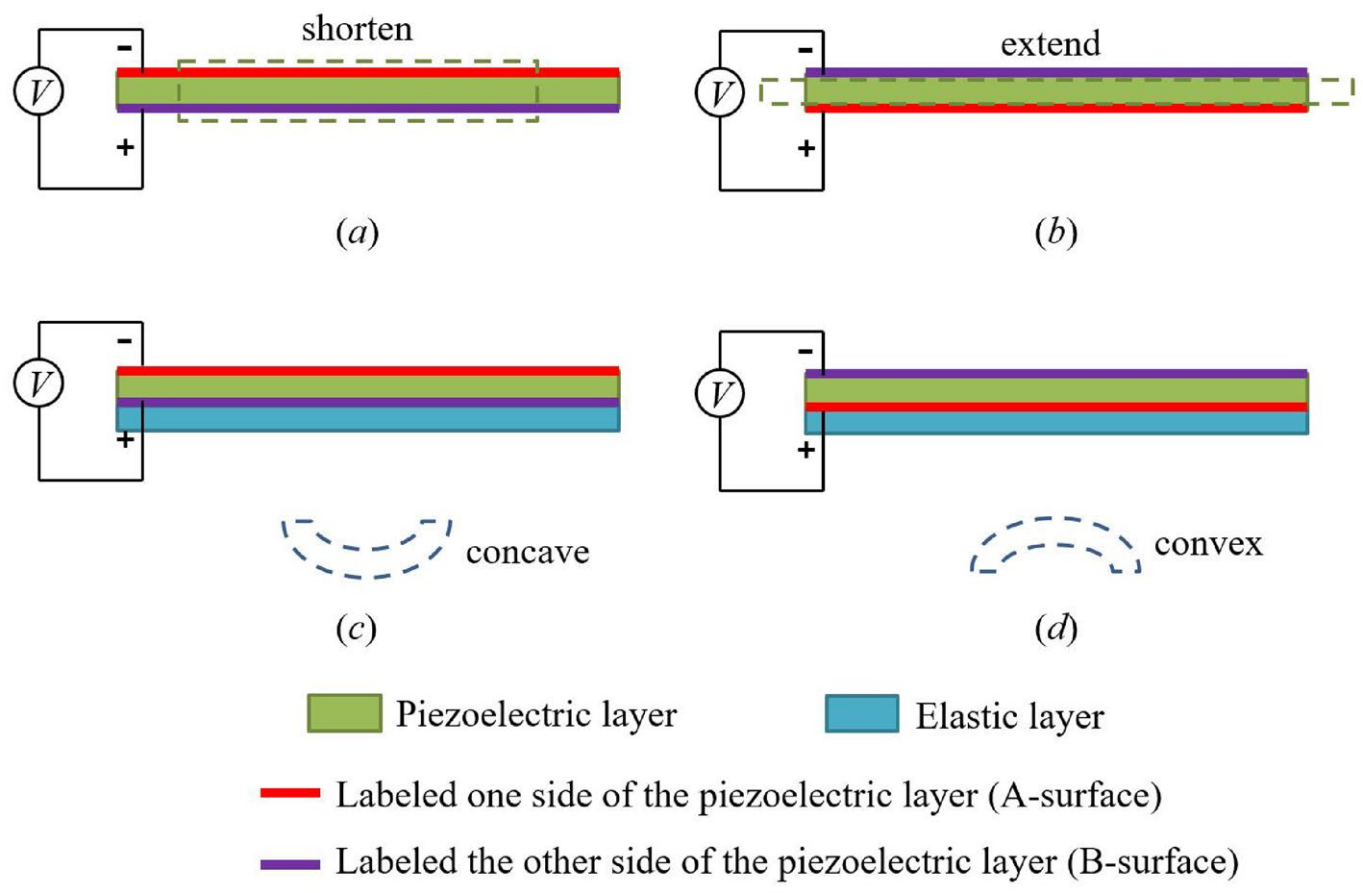
Direction of deformation of piezoelectric layer.

As shown in Fig. 2, a piezoelectric layer is driven by a voltage with positive potential being applied to its lower surface and negative potential being applied to its upper surface. It is well known as Fig. 2a,b that the deformation of the piezoelectric layer will be reversed if we turn the piezoelectric layer over. And when an elastic layer is bonded to the lower surface of the piezoelectric layer, the reversed bending deformation and strain gradient will be generated by turning the piezoelectric layer over, as shown Fig. 2c,d. However, the flexoelectric effect is independent of whether the piezoelectric layer is turned over or not. This means that the flexoelectric effect can both enhance and weaken the electro-mechanical coupling response of the piezoelectric layer by adjusting the orientation of the piezoelectric layer.

**Figure 3:**
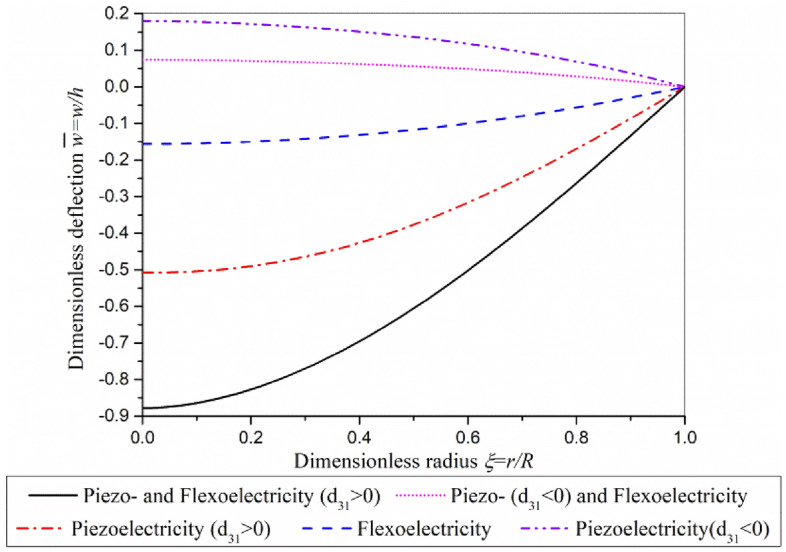
Superposition of piezoelectric effect and flexoelectric effect.

The electro-mechanical coupling superposition of piezoelectric effect and flexoelectric effect is shown in Fig. 3. The bending direction of micro-actuator induced by flexoelectric effect depends on the driving volage. When the driving voltage remains constant, the direction of bending deformation of the actuator is constant, independent of the flip of the piezoelectric layer. Therefore, in one case the flexoelectric effect weakens the electro-mechanical coupling response of the piezoelectric layer (*d*_31_ < 0), then the flexoelectric effect will enhance the electro-mechanical coupling response when the piezoelectric layer is turned over (*d*_31_ > 0) since the direction of bending deformation induced by piezoelectric effect will reverse.

**Figure 4:**
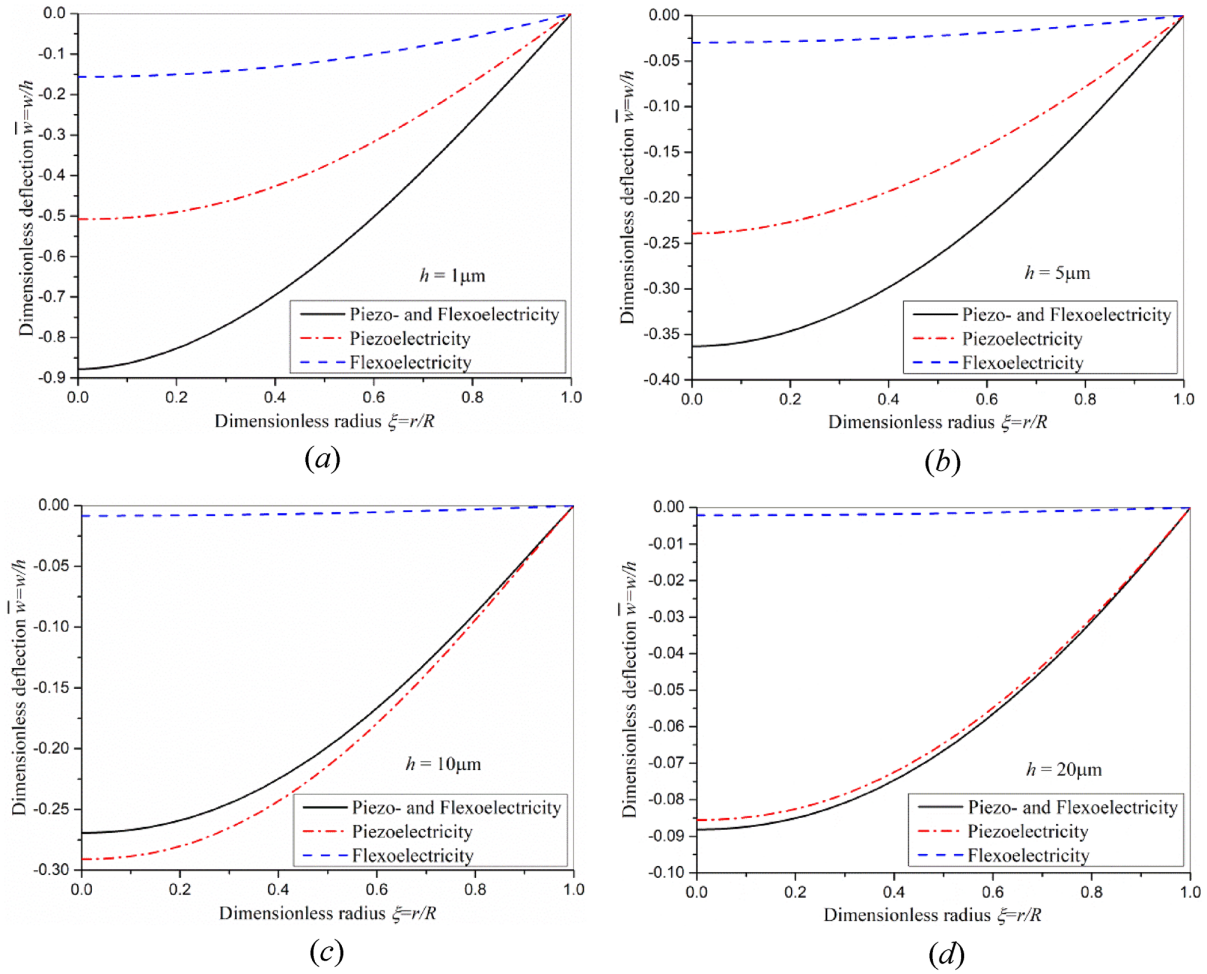
The distribution of induced dimensionless deflection of piezoelectric actuator subjected to a voltage *V* = 80 V with different thicknesses (**a**) *h* = 1 μm (**b**) *h* = 5 μm (**c**) *h* = 10 μm (**d**) *h* = 20 μm.

Under the driving voltage of 80 V, the dimensionless deformation deflection of the piezoelectric micro-actuator of different thicknesses is shown in Fig. 4. It can be found from Fig. 4 that the difference of induced dimensionless deflection by piezoelectric effect, flexoelectric effect and the superimposed effect of these two effects is obvious when the thickness of the actuator is 1 μm. However, with the increase of thickness, the flexoelectric effect gradually weakens and the gap of dimensionless deflection induced by piezoelectric effect and superposition effect gradually shrinks. The flexoelectric effect is size-dependent and can be almost negligible when the actuator thickness is 20 μm or larger. But when the actuator thickness is close to 1 micron or smaller, the contribution of flexoelectric effect is too large to be ignored.

From Eq. (2), the strain on the substrate surface is expressed as

39 $$ \varepsilon_{rr} |_{{z = h_{s} }} = \vartheta \frac{{\partial \overline{u}}}{\partial \xi } + \frac{1}{2}\left( {\vartheta \frac{{\partial \overline{w}}}{\partial \xi }} \right)^{2} - (1 - \alpha )\vartheta^{2} \frac{{\partial^{2} \overline{w}}}{{\partial \xi^{2} }}\quad \varepsilon_{\theta \theta } |_{{z = h_{s} }} = \vartheta \frac{{\overline{u}}}{\xi } - (1 - \alpha )\vartheta^{2} \frac{{\partial \overline{w}}}{\xi \partial \xi } $$

**Figure 5:**
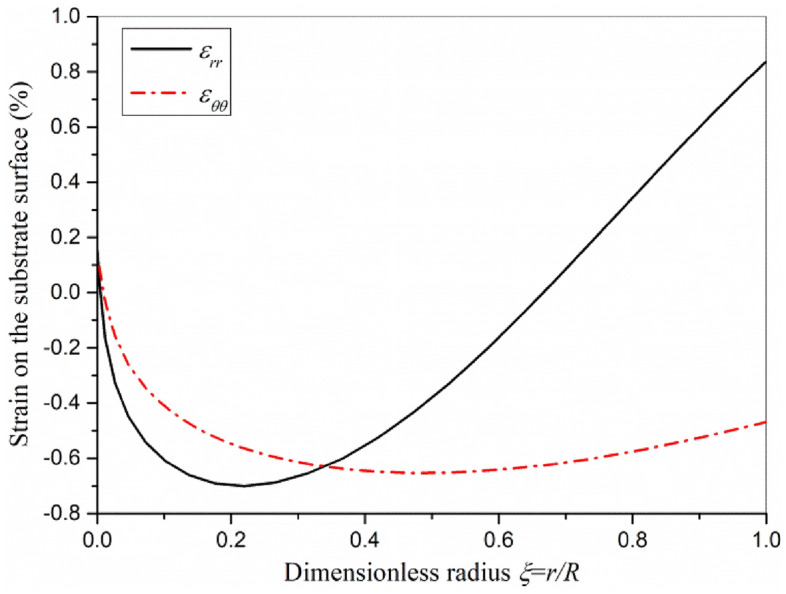
Strain distribution on the substrate surface.

Based on Eq. (39), the strain distribution is shown in Fig. 5. The deformation of the whole actuator plate is the upward convex bending deformation. The circumferential strain is compressive strain. The axial strain near the actuator plate center is compressive strain, and the axial strain near the actuator plate edge is tensile strain. Understanding the strain distribution of actuator on surface will be of great interest for those applications which need stretching stress stimulation.

### Clamped boundary condition

For the clamped circular plate piezoelectric micro-actuator, the governing equations are the same with those of simply supported circular plate piezoelectric micro-actuator and have been discretized as Eqs. (35) and (36). The boundary conditions are

40 $$ \overline{u}|_{\xi = 1} = 0,\quad \overline{w}|_{\xi = 1} = 0,\quad \overline{w}_{,\xi } |_{\xi = 1} = 0 $$

and the regularity conditions are the same with Eq. (33). According to DQM, the boundary conditions are discretized as

41 $$ \overline{u}_{1} = 0,\quad \overline{u}_{N} = 0,\quad \sum\limits_{j = 1}^{N} {A_{1j}^{(1)} \overline{w}_{j} } = 0,\quad \overline{w}_{N} = 0,\quad \sum\limits_{j = 1}^{N} {A_{Nj}^{(1)} \overline{w}_{j} } = 0 $$

**Figure 6:**
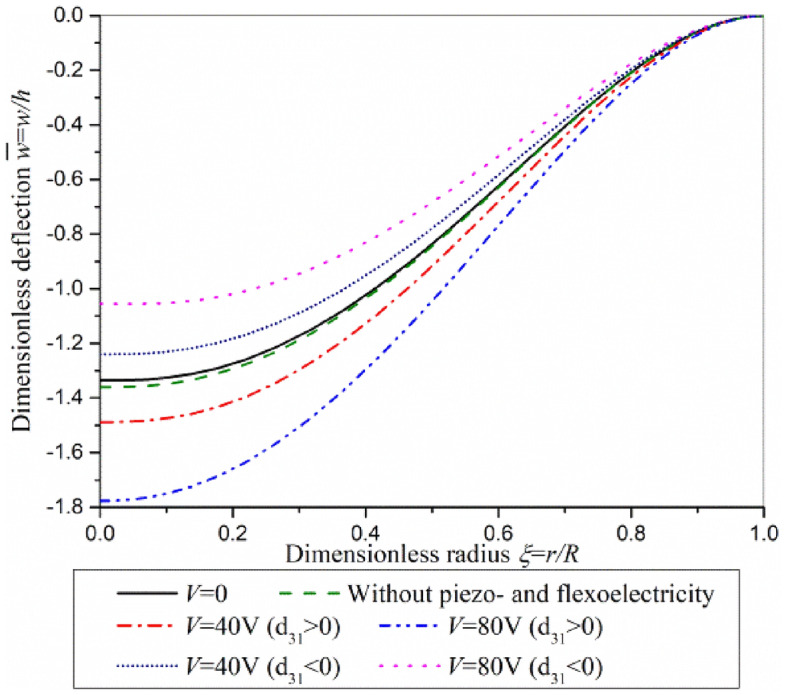
The dimensionless bending deflection of actuator plate subjected to different driving voltage under clamped boundary condition.

The dimensionless bending deflection of actuator plate subjected to transverse load *q* = − 0.3 μN/μm^2^ and different driving voltages under clamped boundary condition are shown as Fig. 6. By comparing the case of *V* = 0 and the case without considering the piezoelectric and flexoelectric effects, it can be found that the piezoelectric and flexoelectric effects will reduce the deformation of the plate but the change is small. The bending deflection induced by the driving voltage increases with the increase of the voltage, but reversed deformation deflection can be induced by a same driving voltage when the piezoelectric layer is turned over. Therefore, the driving voltage-induced deformation increases or decreases the bending deflection generated by the transverse load.

**Figure 7:**
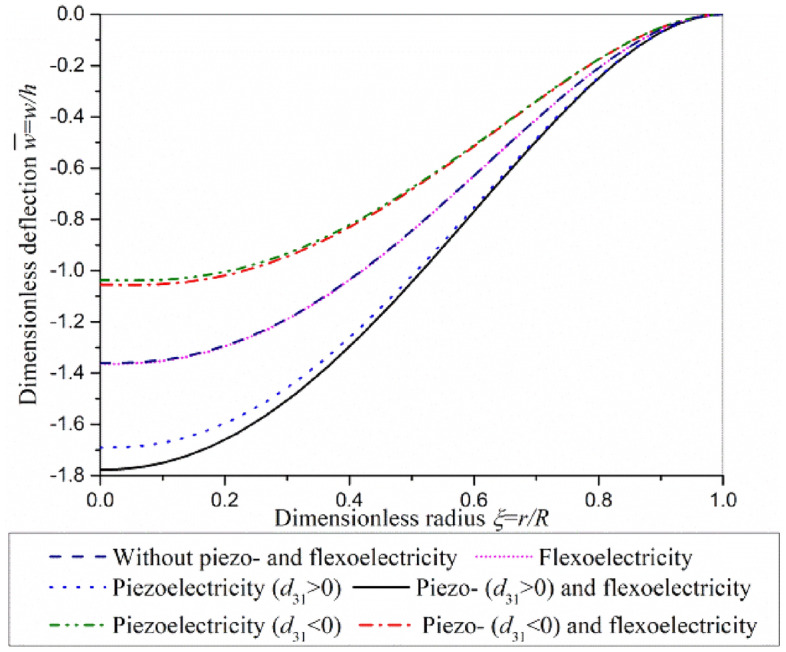
Superposition of piezoelectric effect and flexoelectric effect in actuator plate subjected to *q* = − 0.3 μN/μm^2^ and *V* = 80 V under clamped boundary condition.

The contribution of piezoelectric effect and flexoelectric effect on the bending deflection of actuator under clamped boundary condition is investigated in Fig. 7. The contribution of flexoelectric effect on bending deflection is small in comparison with that of piezoelectric effect. Governing equations and boundary conditions, Eq. (27)–(31), show that the driving voltage which induces deformation through flexoelectric effect acts on the boundary of actuator plate, while the driving voltage which induces deformation through piezoelectric effect acts on the whole actuator plate. For clamped actuator plate, the driving voltage cannot work through flexoelectric effect.

**Figure 8:**
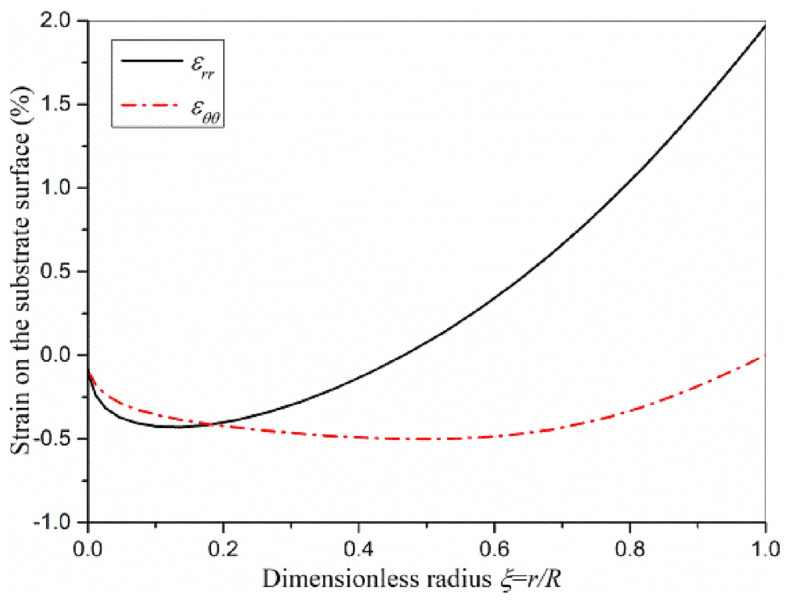
Strain distribution on the substrate surface of actuator plate under clamped boundary condition.

The strain distribution on the substrate surface of actuator plate under clamped boundary condition is shown in Fig. 8. Compared Fig. 8 with Fig. 5, it can be found that the distribution of circumferential strain and axial strain on the substrate surface of actuator plate under simply supported and clamped boundary conditions is similar, but the clamped actuator plate has greater strain because of the transverse load.

## Conclusions

In this paper, the large deflection bending of circular piezoelectric micro-actuator including flexoelectric effect is analyzed based on the extended linear theory for dielectrics and Von Kármán strain theory. A size-dependent model of circular piezoelectric actuator is established by considering the effects of strain gradient and polarization gradient. The corresponding governing equations and boundary conditions are derived based on the variational principle. Boundary value problems of simply supported plate and clamped plate are solved by using the differential quadrature method. The coupling of piezoelectric effect and flexoelectric effect is investigated by the generated large deflection for both cases.

Results show that the orientation of piezoelectric layer affects the superposition of piezoelectric effect and flexoelectric effect. If the flexoelectric effect weakens the electromechanical coupling response of the piezoelectric layer in one case, then the flexoelectric effect will enhance the electromechanical coupling response when the piezoelectric layer is turned over since the direction of bending deformation induced by piezoelectric effect will reverse. Furthermore, the flexoelectric effect is size-dependent. When the actuator thickness is close to 1 micron or smaller, the contribution of flexoelectric effect is too large to be ignored. However, the flexoelectric effect gradually weakens with the increase of thickness and can be almost negligible when the actuator thickness is 20 μm or larger.

### Supplementary Information

Supplementary Information.

## Data Availability

The datasets used and/or analysed during the current study available from the corresponding author on reasonable request.
