# Supplementary material for: Large deflection analysis of circular piezoelectric micro-actuator with flexoelectric effect
Source: Sci Rep. 2023 Nov 8;13:19388. doi: 10.1038/s41598-023-45990-8 (PMC10632473; doi:10.1038/s41598-023-45990-8)
Supplement: Supplementary file 1 — Supplementary Information. [file 41598_2023_45990_MOESM1_ESM.docx]

# Appendix A

*S*(*r*) in Eq. is given as

$\begin{matrix} S(r)=\frac{1}{2}(c_{11}^{s}h_{s}+c_{11}^{p}h_{p})[(\frac{\partial u}{\partial r})^{2}+(\frac{u}{r})^{2}+\frac{1}{4}(\frac{\partial w}{\partial r})^{4}+\frac{\partial u}{\partial r}(\frac{\partial w}{\partial r})^{2}]+\frac{1}{6}(c_{11}^{s}h_{s}^{3}+c_{11}^{p}h_{p}^{3})[(\frac{\partial^{2}w}{\partial r^{2}})^{2}+(\frac{\partial w}{r\partial r})^{2}] \\ +\frac{1}{4}(c_{11}^{p}h_{p}^{2}-c_{11}^{s}h_{s}^{2})[2\frac{u}{r^{2}}\frac{\partial w}{\partial r}+2\frac{\partial u}{\partial r}\frac{\partial^{2}w}{\partial r^{2}}+(\frac{\partial w}{\partial r})^{2}\frac{\partial^{2}w}{\partial r^{2}}]+(c_{12}^{p}h_{p}+c_{12}^{s}h_{s})[\frac{u}{r}\frac{\partial u}{\partial r}+\frac{1}{2}\frac{u}{r}(\frac{\partial w}{\partial r})^{2}] \\ +\frac{1}{2}(c_{12}^{p}h_{p}^{2}-c_{12}^{s}h_{s}^{2})[\frac{\partial w}{r\partial r}\frac{\partial u}{\partial r}+\frac{1}{2r}(\frac{\partial w}{\partial r})^{3}+\frac{u}{r}\frac{\partial^{2}w}{\partial r^{2}}]+\frac{1}{3}(c_{12}^{p}h_{p}^{3}+c_{12}^{s}h_{s}^{3})\frac{\partial^{2}w}{\partial r^{2}}\frac{\partial w}{r\partial r} \\ +\frac{1}{2}(g_{11}^{p}h_{p}+g_{11}^{s}h_{s})[(\frac{\partial^{2}w}{\partial r^{2}})^{2}+(\frac{\partial w}{r\partial r})^{2}]+(g_{12}^{p}h_{p}+g_{12}^{s}h_{s})\frac{\partial^{2}w}{\partial r^{2}}\frac{\partial w}{r\partial r} \end{matrix}$ (A.1)

The dimensionless coefficients *k_i_*(*i*=1,2,…,8) of the piezoelectric micro-actuator model in Eqs.- are given by

$k_{1}=\frac{(c_{12}^{p}h_{p}+c_{12}^{s}h_{s})-(c_{11}^{s}h_{s}+c_{11}^{p}h_{p})}{2(c_{11}^{s}h_{s}+c_{11}^{p}h_{p}-t_{3})}$ (A.2)

$k_{2}=\frac{2t_{2}-(c_{11}^{p}h_{p}^{2}-c_{11}^{s}h_{s}^{2})}{2R(c_{11}^{s}h_{s}+c_{11}^{p}h_{p}-t_{3})}$ (A.3)

$k_{3}=\frac{2c_{11}^{s}h_{s}^{3}+2c_{11}^{p}h_{p}^{3}+6g_{11}^{p}h_{p}+6g_{11}^{s}h_{s}+6t_{1}}{3h^{2}(c_{11}^{s}h_{s}+c_{11}^{p}h_{p}-t_{3})}$ (A.4)

$k_{4}=\frac{c_{12}^{p}h_{p}+c_{12}^{s}h_{s}-t_{3}}{c_{11}^{s}h_{s}+c_{11}^{p}h_{p}-t_{3}}$ (A.5)

$k_{5}=\frac{c_{11}^{p}h_{p}^{2}-c_{11}^{s}h_{s}^{2}-2t_{2}}{h(c_{11}^{s}h_{s}+c_{11}^{p}h_{p}-t_{3})}$ (A.6)

$k_{6}=\frac{(c_{11}^{p}h_{p}^{2}-c_{11}^{s}h_{s}^{2})-3(c_{12}^{p}h_{p}^{2}-c_{12}^{s}h_{s}^{2})+4t_{2}}{(c_{11}^{s}h_{s}+c_{11}^{p}h_{p}-t_{3})h}$ (A.7)

$k_{7}=\frac{c_{12}^{p}h_{p}^{2}-c_{12}^{s}h_{s}^{2}-2t_{2}}{h(c_{11}^{s}h_{s}+c_{11}^{p}h_{p}-t_{3})}$ (A.8)

$k_{8}=\frac{c_{12}^{p}h_{p}^{3}+c_{12}^{s}h_{s}^{3}+3g_{12}^{p}h_{p}+3g_{12}^{s}h_{s}+3t_{1}}{3h^{2}(c_{11}^{s}h_{s}+c_{11}^{p}h_{p}-t_{3})}$ (A.9)
